# An Investigation of the Non-selective Etching of Synthetic Polymers by Electrospray Droplet Impact/Secondary Ion Mass Spectrometry (EDI/SIMS)

**DOI:** 10.5702/massspectrometry.A0114

**Published:** 2023-01-31

**Authors:** Kenzo Hiraoka, Yuji Sakai, Hiroyuki Kubota, Satoshi Ninomiya, Stephanie Rankin-Turner

**Affiliations:** 1 Clean Energy Research Center, University of Yamanashi, 4–3–11 Takeda, Kofu, Yamanashi 400–8511, Japan; 2 Department of Molecular Microbiology & Immunology, Johns Hopkins Bloomberg School of Public Health, Johns Hopkins University, 615 N. Wolfe Street, Baltimore, MD, 21205, USA

**Keywords:** EDI/SIMS, cluster SIMS, polystyrene, poly(9,9-di-*n*-octylfluonyl-2,7diyl)

## Abstract

Among the various types of cluster secondary ion mass spectrometry (SIMS), electrospray droplet impact/secondary ion mass spectrometry (EDI/SIMS) is unique due to its high ionization efficiency and non-selective atomic/molecular-level surface etching ability. In this study, EDI/SIMS was applied to the non-selective etching of synthetic polymers of polystyrene (PS) and poly(9,9-di-*n*-octylfluonyl-2,7diyl) (PFO) deposited on a silicon substrate. The polymers gave characteristic fragment ions and the mass spectra remained unchanged with prolonged EDI irradiation time, indicating that non-selective etching can be achieved by EDI irradiation, a finding that is consistent with our previous reports based on EDI/X-ray photoelectron spectroscopy analyses. From the irradiation time and film thickness, the etching rates for PS and PFO were roughly estimated to be 0.6 nm/min and 0.15 nm/min, respectively, under the experimental conditions that were used. After the depletion of polymer sample on the surface, ion signals originating from the exposed silicon substrate were observed. This indicates that EDI/SIMS is applicable to the analysis of the interface of multilayered films composed of organic and inorganic materials.

## INTRODUCTION

Secondary ion mass spectrometry (SIMS) using high-speed projectiles for the sputtering/ionization of the sample is one of the most powerful techniques for the characterization of many materials. The injection of atomic projectiles into solid materials results in linear cascade collisions and inevitably results in damage under the surface. A number of studies using cluster ions as projectiles, termed cluster SIMS, have shown that the sputtering and the efficiency of secondary ion formation improves with increasing mass of the primary particles.^1–15)^ Various types of molecular and cluster ions, including Cs^+^ (CsI)*_n_*,^1)^ SF_5_^+^,^12)^ Au*_n_*^+^,^10)^ Bi*_n_*^+^,^16,17)^ C_60_^+^,^8)^ Ar*_n_*^+^^18)^ and massive glycerol clusters have been developed.^19–22)^ Among these, the formation of massive, multiple-charged glycerol clusters is considered to be particularly advantageous. Massive multiple-charge glycerol clusters with masses of 10^6^–10^7^ u and excess charges of ∼200 can be generated *via* electrohydrodynamic emission under a vacuum using a 1.5 M solution of ammonium acetate in glycerol.^19–22)^ This method of massive cluster impact (MCI) has enabled the production of high yields of secondary-ion from biological samples without the need for matrices. Although MCI was found to afford extremely soft ionization/desorption conditions for peptides and proteins,^19–22)^ a stable ion current can only be obtained for several hours until the lens electrodes become contaminated by accumulated glycerol. In our previous studies,^23–25)^ we reported that this problem can be solved by using a more volatile solvent, which led to the development of the electrospray droplet impact (EDI) technique that uses the atmospheric pressure electrospray of acidic aqueous solvent instead of the vacuum electrospray of glycerol. It was found that EDI has unique characteristics that include (1) an extremely high ionization efficiencies compared to other cluster SIMS techniques,^26,27)^ (2) surface cleaning effect due to the ability of atomic and molecular-level etching,^23–25)^ (3) non-selective etching for organic and inorganic materials,^28)^ (4) the ability to ionize both organic and inorganic materials (*e.g.*, Au, In, Si, SiO_2_),^28,29)^ (5) long-term maintenance-free operation.^28)^

The ability to achieve non-selective etching is of particular importance for the application of EDI to the analysis of the interface between multilayered samples. In our laboratory, interface analysis mainly used EDI coupled with X-ray photoelectron spectroscopy (EDI/XPS).^30–33)^ For example, XPS using Ar^+^ ions as projectiles is difficult to apply to the interface analysis of CuO/Cu because the selective etching of oxygen in the CuO layer occurs and CuO is reduced to Cu by the Ar^+^ bombardment.^30)^ In contrast, the interface of CuO/Cu could be clearly defined by EDI/XPS due to the non-selective etching of EDI.^30)^

Interface analysis using EDI/SIMS has not been sufficiently explored in comparison to EDI/XPS. In this study, we report on the application of EDI/SIMS to the analysis of bilayer samples of polystyrene/Si and poly(9,9-di-*n*-octylfluonyl-2,7diyl)/Si. The results indicated that the mass spectra for synthetic polymers did not change with EDI irradiation time. This result is in excellent agreement with data obtained using EDI/XPS. It was also found that the results obtained by EDI/SIMS and EDI/XPS are complementary to each other.

## EXPERIMENTAL

### Materials and sample preparation

Reagent grade toluene and chloroform were purchased from FUJIFILM Wako Pure Chemical (Osaka, Japan). Films of polystyrene (PS) and poly(9,9-di-*n*-octylfluonyl-2,7diyl) (PFO) with a 0.05 mm thickness were purchased from Goodfellow (Cambridgeshire, England). 2 wt% of PS in toluene and 0.5% of PFO in chloroform were prepared for spin coating. The samples were coated on the HF-treated native silicon substrate (100) by using a spin coater (SPN-T02HV, Sanyu Electron Co. Ltd., Tokyo, Japan) operated at 5000 rpm for 60 s and annealed at 90°C for 5 min. The thicknesses of the PS and PFO were measured by a spectroscopic ellipsometer (SE 800, Sentech, Berlin, Germany) as 38 nm and 22 nm, respectively. The surface roughness of the spin-coated PS film was measured by atomic force microscopy (JSPM-5400, JEOL, Akishima, Japan) as 0.2∼0.3 nm.

### Mass spectrometric measurements

The conceptual idea of an EDI/SIMS apparatus is shown in Fig. S1.^23,28)^ The charged liquid droplets are formed by electrospraying a 1 M aqueous solution of acetic acid with a flow rate of 50 μL min^−1^. The voltages applied to the stainless steel capillary (i.d.: 0.1 mm, o.d.: 0.2 mm) were +3 kV. The electrospray was nebulized with N_2_ gas at a flow rate of ∼8 L min^−1^. From the N_2_ gas flow rate and i.d. of the capillary (0.3 mm) for nebulization, the linear velocity of the nebulizing gas through the tip nozzle was estimated to be roughly ∼400 m s^−1^. The charged liquid droplets were sampled through a 400 μm diameter orifice into the first vacuum chamber, skimmed through a second orifice with a 1.2 mm diameter, and transported into a first quadrupole ion guide which was adjusted to transport charged droplets in the *m*/*z* range of 1×10^4^−5×10^5^. The pressures of the first vacuum chamber (between orifice 1 and orifice 2), the first ion guide, the sample stage, and the second ion guide, were 4×10^−1^, 5×10^−4^, 2×10^−5^, and 1×10^−4^∼3×10^−3^ Pa, respectively. By assuming that the charged droplets are close to the Rayleigh limit, the masses of the droplets and the number of charges were estimated to be in the range of 6.2×10^5^ to 1.6×10^7^ u, and 62−311, respectively. The radii of the droplets was calculated to be 5−20 nm. After exiting the ion guide, the charged droplets were accelerated by 10 kV and impacted the target (ground potential) at an incident angle of 60° to the surface normal. The beam diameter was about 3 mm with a blurred peripheral region as shown in Fig. S1(b). The kinetic energy and velocity of a typical projectile [(H_2_O)_90,000_ + 100H]^100+^ accelerated by 10 kV was calculated to be 1×10^6^ eV and 12 km s^−1^, respectively. The velocity of droplets is about one order of magnitude larger than the sound velocity of the solids (∼a few km/s). Thus, a shock wave should be generated in the colliding interface between the water droplet and the solid sample. As a result of the supersonic collision, an enormous high pressure is generated at the moment of collision.^19–22)^ Due to the instant high pressure built-up at the colliding interface, the heterogeneous ionization of water molecules (2H_2_O → H_3_O^+^ + OH^−^) at the colliding interface occurs, *e.g.*, the formation of reactant ions such as H_3_O^+^ and OH^−^ that act as protonating and deprotonating reagent ions, respectively.^28)^

The secondary ions formed by impact of the projectile droplet are transported into a second quadrupole ion guide for collisional cooling and mass-analysis by an orthogonal time-of-flight mass spectrometer (AccuTOF, JEOL, Akishima, Japan). The nascent cluster ions generated by EDI suffered from multiple collisions with the N_2_ buffer gas in the second ion guide and more stable cluster ions were preferentially formed by the degradation of larger cluster ions. The measurements were made by the pulse-counting method using a 4 GHz TOF multiscaler (P7887, FAST ComTec GmbH, Oberhaching, Germany).

## RESULTS AND DISCUSSION

Although EDI/SIMS has been used for the detection of proteins such as cytochrome *c* (MW: 12327) as intact forms,^24)^ fragment ions are observed as the major ions for organic compounds with MWs larger than a few 10^4^. This is the typical case for industrial synthetic polymers. In such cases, various fragment ions are observed by EDI bombardment. It is thought that selective etching occurs during the prolonged EDI irradiation, *e.g.*, the selective etching of H, O and N elements resulting in the graphitization of the samples. As described above, such a segregation of elements has not been observed for synthetic polymers studied by EDI/XPS. In this work, EDI/SIMS was used to examine the occurrence or non-occurrence of selective etching using PS and PFO as model samples.

### Polystyrene (PS)

**Figure 1:**
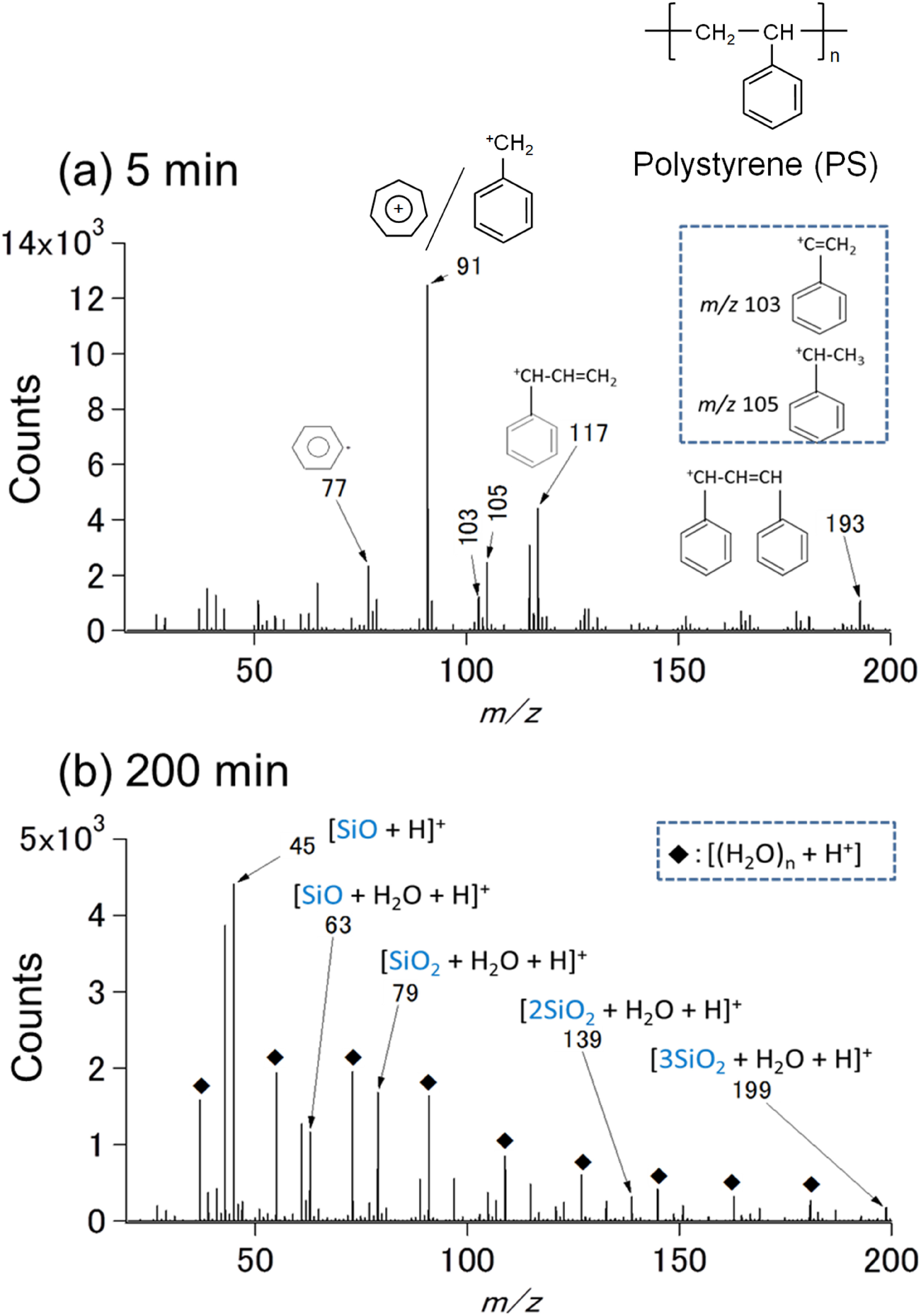
Fig. 1. Mass spectra of PS (38 nm) spin-coated on a Si substrate. (a) EDI irradiation time: 5 min. (b) EDI irradiation time: 200 min.

Figure 1 shows mass spectra obtained at 5 min and 200 min after the start of the EDI irradiation of the 38 nm thick PS film deposited on the Si substrate. As shown in Fig. 1(a) a 5 min irradiation by EDI produced several characteristic fragment ions for PS, suggesting that EDI/SIMS may be useful for the characterization of synthetic polymers. After 200 min of EDI irradiation as shown in Fig. 1(b), fragment ions originating from PS are barely detected and, instead, ions originating from the exposed Si appeared. The appearance of [SiO+H]^+^ and [SiO+H_2_O+H]^+^ indicates that the substrate surface was composed of pristine Si.^34)^ This shows that the oxide layer of the native silicon substrate could be removed by the HF treatment. In Fig. 1(b), cluster ions containing proton-bound water molecules were detected that are absent in Fig. 1(a). This suggests that not all of the reactant ions of H_3_O^+^ that are generated at the colliding interface were consumed by the ionization of analytes during the collisional event and that some fraction of the H_3_O^+^ ions form cluster ions with environmental H_2_O vapor. In contrast, as shown in Fig. 1(a), almost all the reactant H_3_O^+^ ions reacted with desorbed PS fragments and were annihilated at the colliding interface.

**Figure 2:**
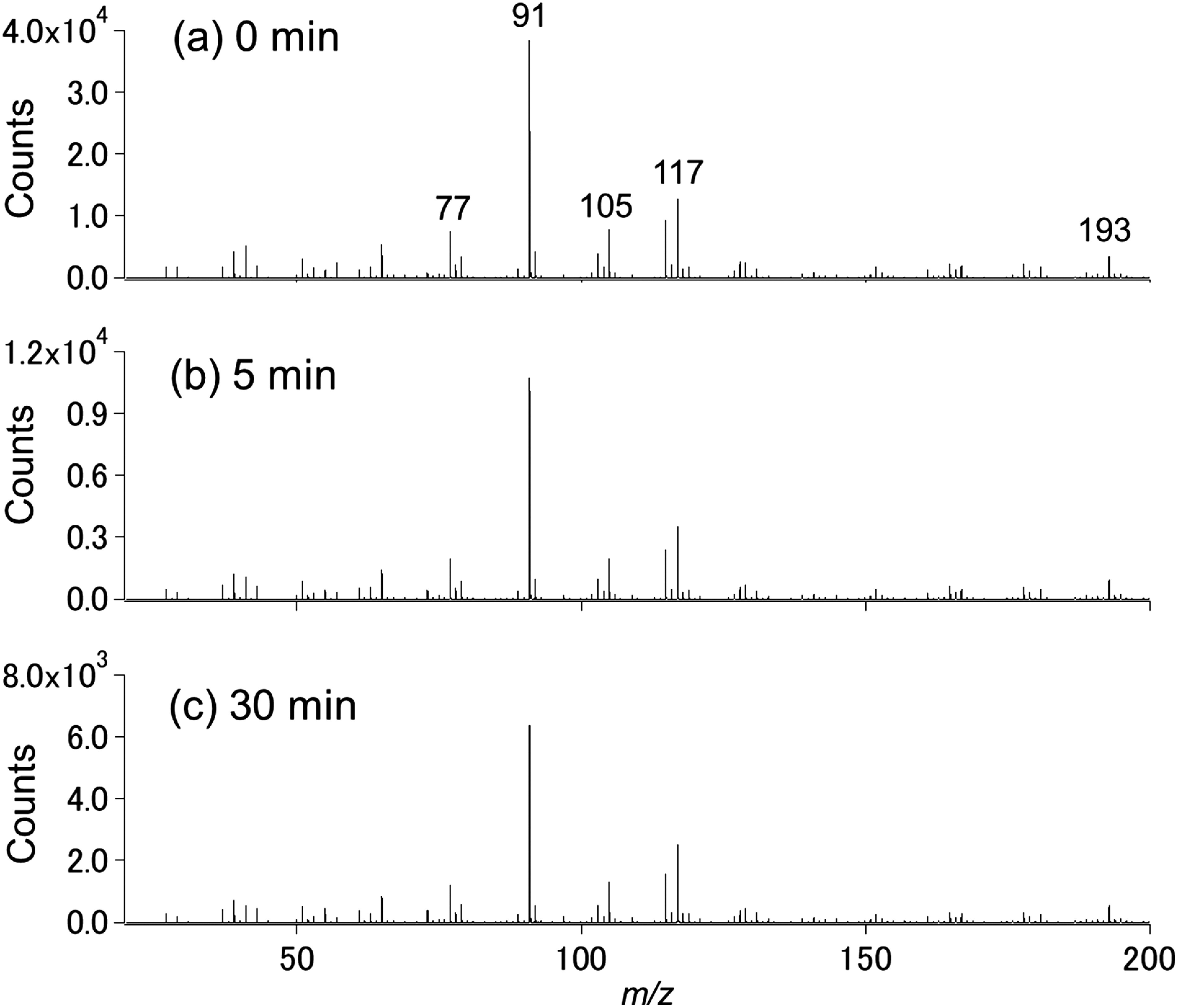
Fig. 2. Mass spectra of PS (38 nm)/Si measured with the EDI irradiation time of (a) 0 min, (b) 5 min, and (c) 30 min.

**Figure 3:**
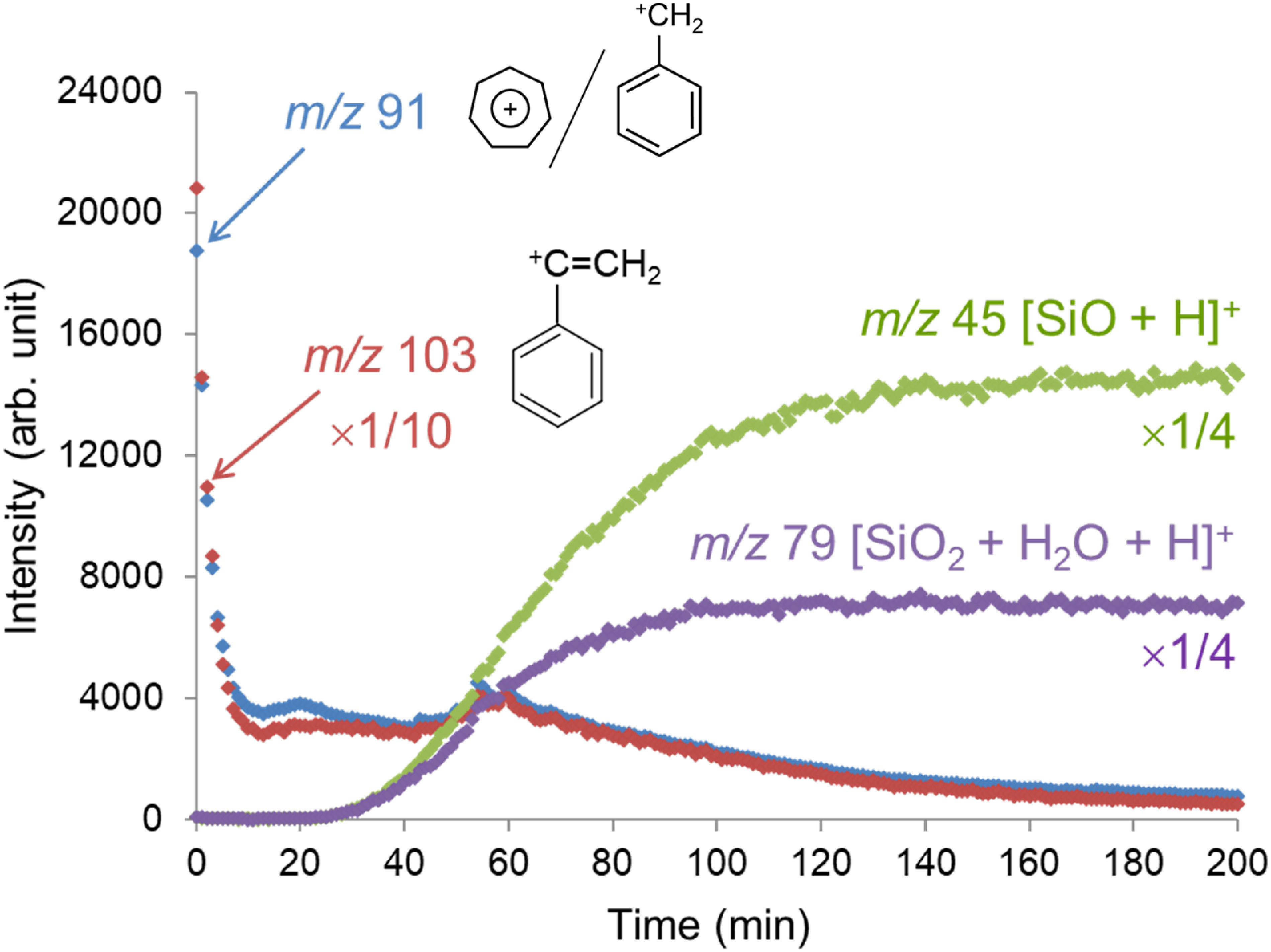
Fig. 3. The dependence of EDI irradiation time on the intensities of C_7_H_7_^+^ (*m*/*z* 91) and C_8_H_7_^+^ (*m*/*z* 103) originating from PS, and [SiO+H]^+^ (*m*/*z* 45) and [SiO_2_+H_2_O+H]^+^ (*m*/*z* 79) originating from the Si substrate for the sample of PS (38 nm)/Si. The intensities of C_8_H_7_^+^ (*m*/*z* 103) were multiplied by 10 and those of [SiO+H]^+^ (*m*/*z* 45) and [SiO_2_+H_2_O+H]^+^ (*m*/*z* 79) were multiplied by 4 for the sake of clarity.

Figure 2 shows mass spectra measured at 0, 5, and 30 min after the start of EDI irradiation. Although the ion intensities decreased with irradiation time, the relative intensities of the fragment ions remained nearly constant. This indicates that the selective etching of elemental hydrogen from the PS sample by EDI is nearly negligible. Figure 3 shows the effect of EDI irradiation time on the intensities of C_7_H_7_^+^ (*m*/*z* 91) and C_8_H_7_^+^ (*m*/*z* 103) originating from PS, and [SiO+H]^+^ (*m*/*z* 45) and [SiO_2_+H_2_O+H]^+^ (*m*/*z* 79) originating from the Si substrate. As shown in Fig. 3, [SiO+H]^+^ (*m*/*z* 45) and [SiO_2_+H_2_O+H]^+^ (*m*/*z* 79) begin to be observed at ∼25 min indicating that the Si surface is partially exposed after the ∼25 min EDI irradiation. Contrary to the nearly constant relative intensities of fragment ions for the PS overlayer, as shown in Fig. 2, the ratio of the intensities of *I*([SiO+H]^+^)/*I*([SiO_2_+H_2_O+H]^+^) in Fig. 3 was found to increase with EDI irradiation time. In our previous report,^34)^ we observed adduct ions of OH^−^ with SiO and H_2_O for pristine Si but not for SiO_2_. The increase in the ratio of *I*([SiO+H]^+^)/*I*([SiO_2_+H_2_O+H]^+^) suggests that the partially oxidized Si surface was being removed by the etching process and that pristine silicon was exposed to the prolonged EDI irradiation. This result suggests that EDI can be applicable for the analysis of a SiO_2_/Si interface, as reported in our previous study.^34)^

In Fig. 3, a rapid decrease in the intensity of C_7_H_7_^+^ (*m*/*z* 91) and C_8_H_7_^+^ (*m*/*z* 103) was observed in the initial 10 min. This can be attributed to the electric charging of the insulating PS film induced by the charged droplet irradiation. Two humps appeared at ∼20 min and ∼55 min after the start of the EDI irradiation. The appearance of these humps may be due to the partial exposure of the underlayer electroconductive Si substrate that moderated the charging of the film. In contrast to PS, electric charging was not observed for the electroconductive sample of PFO (see the next section).

From the film thickness of PS (38 nm) and the EDI irradiation time of ∼60 min at which the ion intensities of C_7_H_7_^+^ (*m*/*z* 91) and C_8_H_7_^+^ (*m*/*z* 103) start to decrease, the etching rate was roughly estimated to be 0.6 nm/min.

The slow decreases in the intensity of C_7_H_7_^+^ (*m*/*z* 91) and C_8_H_7_^+^ (*m*/*z* 103) after 60 min of EDI irradiation accompanied by gradual increases in [SiO+H]^+^ (*m*/*z* 45) and [SiO_2_+H_2_O+H]^+^ (*m*/*z* 79) in Fig. 3 may be mainly due to the blurred ion beam, as shown in Fig. S1 (b) (*i.e.*, a beam edge effect). In fact, a much better interface analysis could be performed by using a beam with a diameter of 0.2 mm scanned over a 4×4 mm^2^ area for CuO/Cu.^30)^

### Poly(9,9-di-*n*-octylfluonyl-2,7diyl) (PFO)

**Figure 4:**
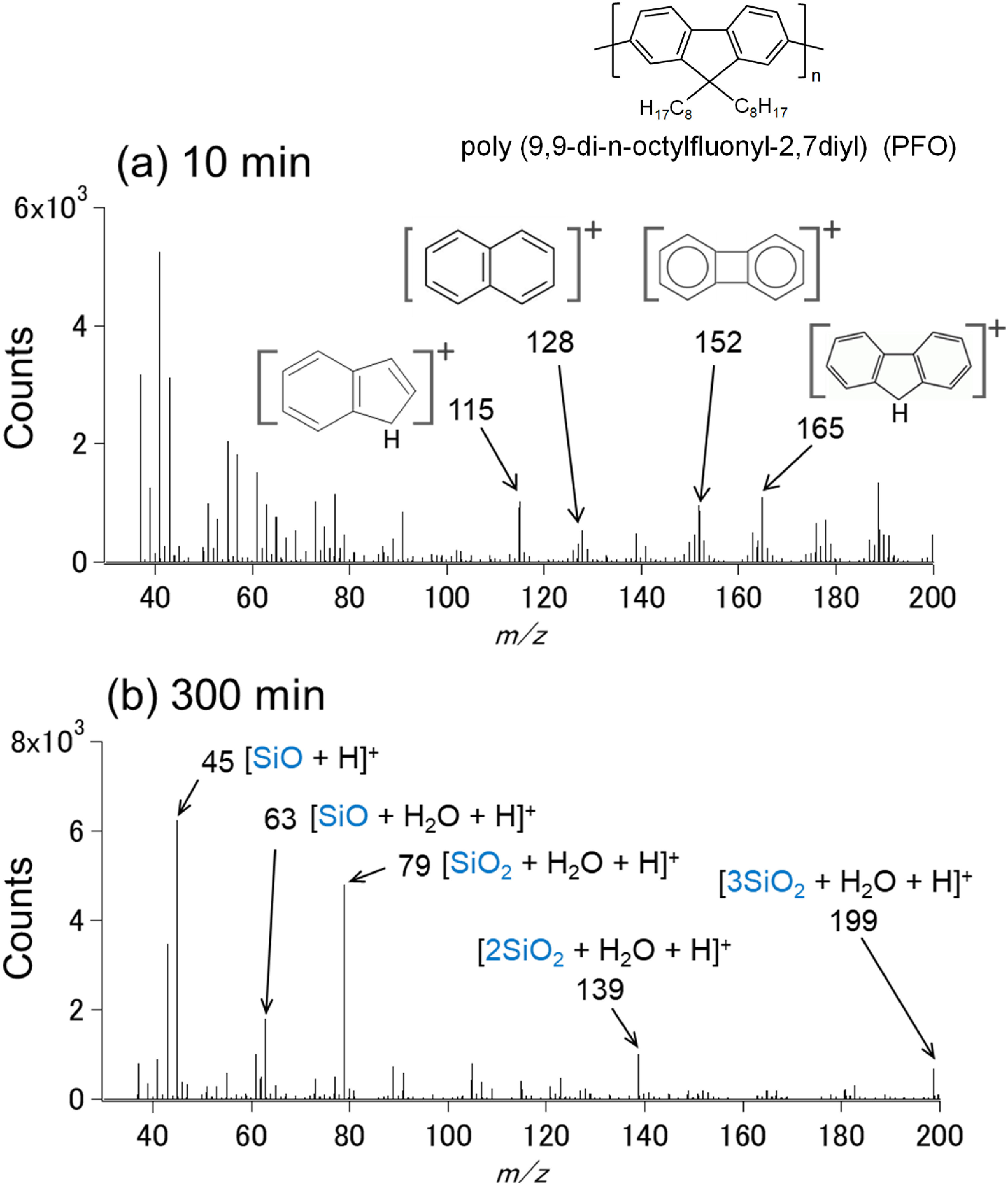
Fig. 4. Mass spectra of PS (38 nm)/Si measured at (a) 10 min and (b) 300 min after the start of EDI irradiation in the *m*/*z* range of 30–200.

Figure 4 shows mass spectra measured at 10 min and 300 min after the start of EDI irradiation in the *m*/*z* range of 30–200. In Fig. 4(a), all of the ions that were detected originated from benzene-ring-linkage backbones and no ions originating from the side aliphatic chains could be identified. Upon EDI irradiation, the aliphatic chains of PFO with sp^3^ hybridization bonds had dissociated and formed more stable aromatic ring structures with sp^2^ hybridization. Thus, the use of EDI/SIMS to the characterization of PFO for the identification of aliphatic chains is not an easy task. In the case of 300 min of EDI irradiation, fragment ions originating from PFO were barely detected and ions originating from the silicon substrate were the most common.

**Figure 5:**
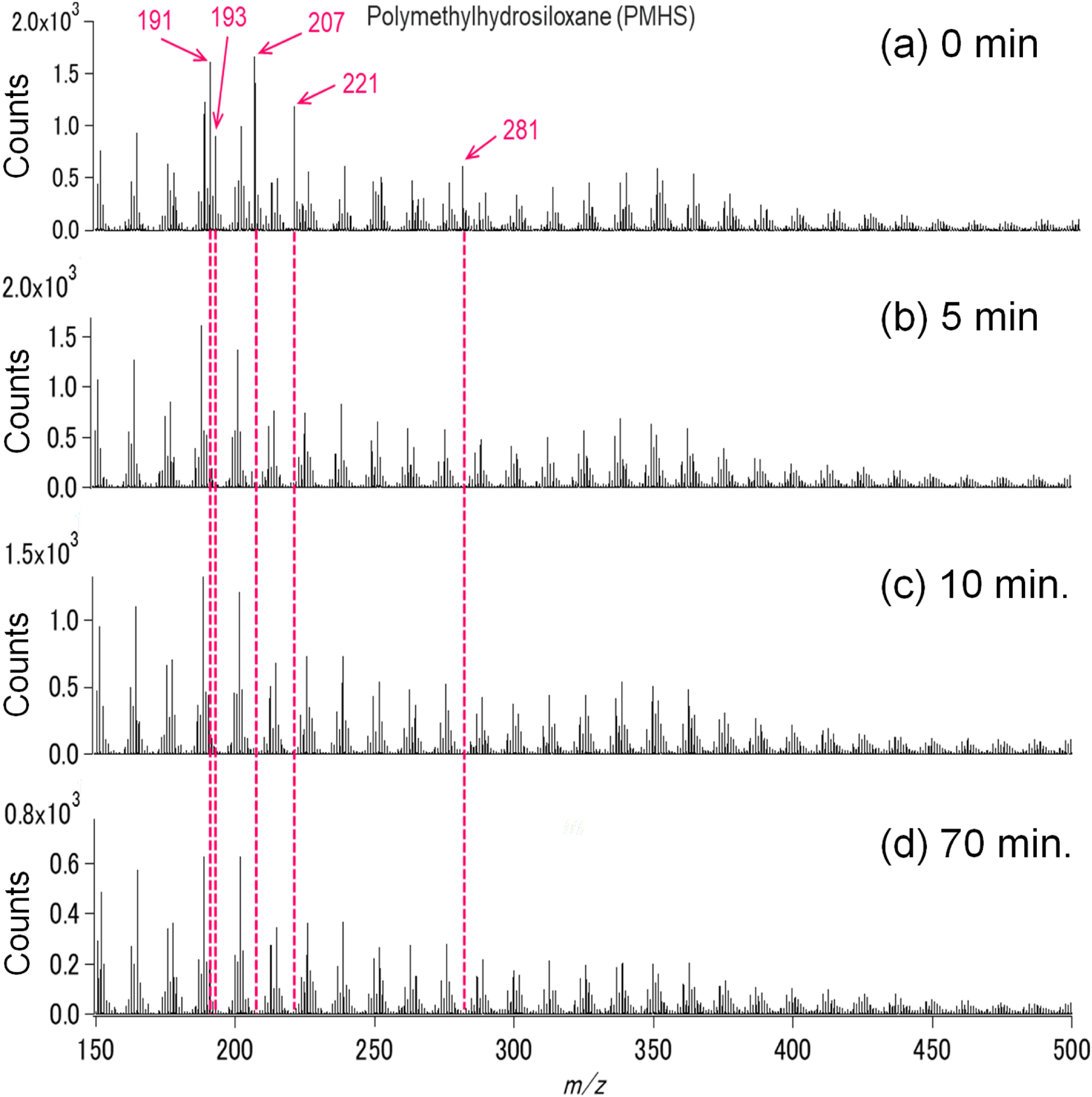
Fig. 5. Mass spectra of PFO (22 nm)/Si measured at (a) 0, (b) 5, (c) 10, and (d) 70 min after the start of EDI irradiation.

**Figure 6:**
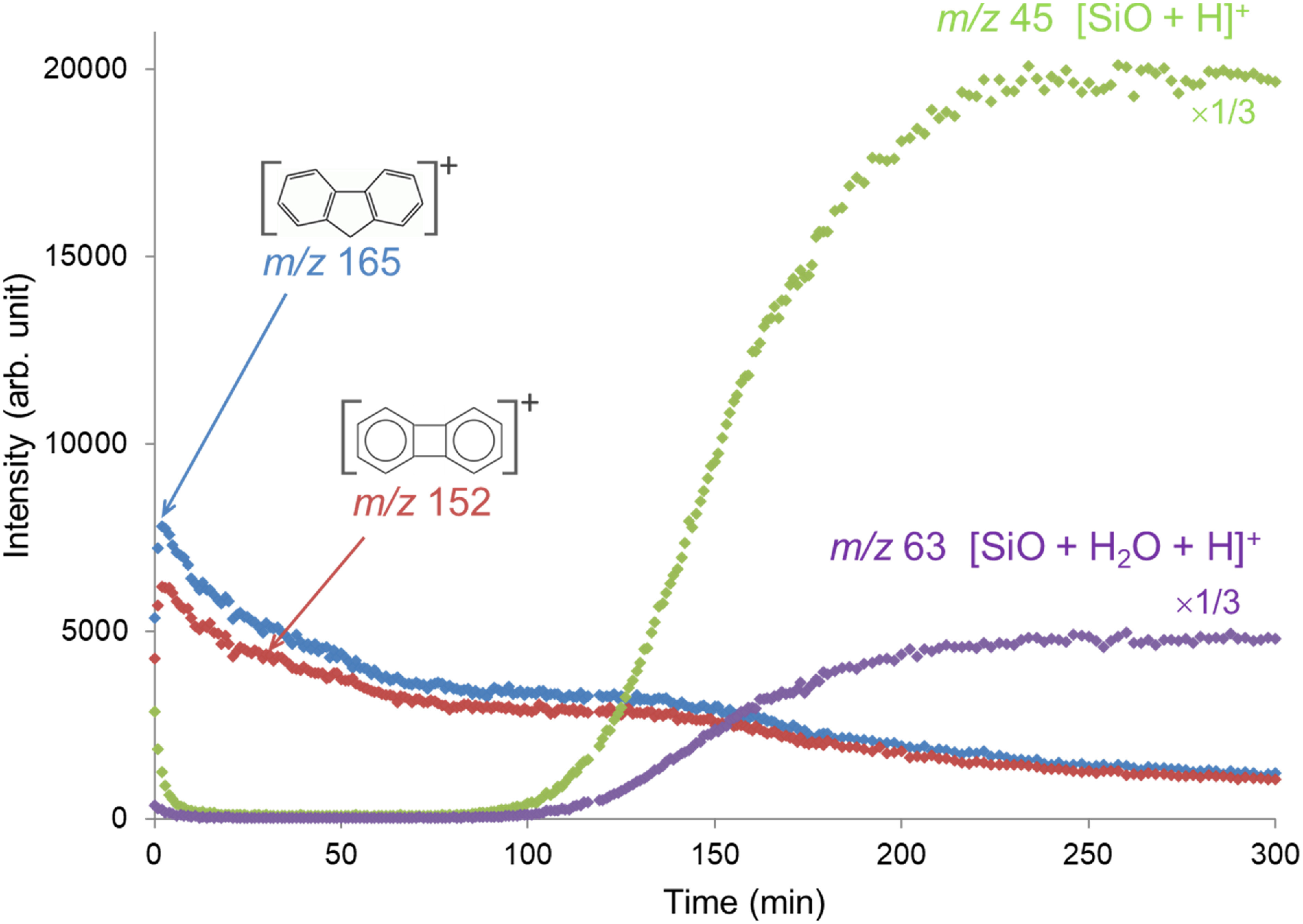
Fig. 6. The dependence of EDI irradiation time on the intensities of C_12_H_8_^+^ (*m*/*z* 152) and C_13_H_9_^+^ (*m*/*z* 165) originating from PFO, and [SiO+H]^+^ (*m*/*z* 45) and [SiO+H_2_O+H]^+^ (*m*/*z* 63) originating from Si substrate for the sample of PFO (22 nm)/Si. The intensities of [SiO+H]^+^ (*m*/*z* 45) and [SiO+H_2_O+H]^+^ (*m*/*z* 63) were multiplied by 3 for the sake of clarity.

Figure 5 shows mass spectra measured at 0, 5, 10, and 70 min after the start of EDI irradiation. At the start of irradiation (0 min), peaks at *m*/*z* 191, 193, 207, 221, and 281 were detected as major ions. From the mass spectral pattern, these peaks are tentatively assigned to polymethylhydrosiloxane (PMHS), an impurity. Because of its high surface activity, it is possible that the surface of PFO film sample may have become contaminated by PMHS during the sample preparation. After a few min of EDI irradiation, these peaks disappeared and the underlayer PFO sample was exposed. It therefore appears that EDI has a surface-cleaning effect due to the atomic- and molecular-level etching ability of this procedure.^23)^ Figure 6 shows the dependence of time of EDI irradiation on the intensities of C_12_H_8_^+^ (*m*/*z* 152) and C_13_H_9_^+^ (*m*/*z* 165) originating from PFO, and [SiO+H]^+^ (*m*/*z* 45) and [SiO+H_2_O+H]^+^ (*m*/*z* 63). The initial sharp drops in the intensity of [SiO+H]^+^ (*m*/*z* 45) and [SiO+H_2_O+H]^+^ (*m*/*z* 63) are likely due to the initial desorption of PMHS segregated on the PFO sample surface. The rapid decrease in the intensity of these ions was accompanied by a sharp increase in C_12_H_8_^+^ (*m*/*z* 152) and C_13_H_9_^+^ (*m*/*z* 165) originating from PFO.

Although an initial sharp decrease in signal intensities was observed for PS (see Fig. 3), the decrease in the intensities of C_12_H_8_^+^ (*m*/*z* 152) and C_13_H_9_^+^ (*m*/*z* 165) in Fig. 6 became much slower with increasing EDI irradiation time. This suggests that the electric charging of the PFO film caused by the EDI irradiation was minor compared to PS. This is reasonable because PFO is an electroconductive substance.

From the film thickness of PFO (22 nm) and an EDI irradiation time of ∼150 min at which the intensities of the C_12_H_8_^+^ (*m*/*z* 152) and C_13_H_9_^+^ (*m*/*z* 165) ions start to decrease, the etching rate was roughly estimated to be 0.15 nm/min. This value is much smaller than that for PS (0.6 nm/min). This can be attributed to the fact that the internal energy imparted to the PFO film by EDI irradiation is dispersed throughout the π-electron delocalized polymer main chain. That is, the density of states for PFO is much denser than that of PS for the energy dissipation. This suggests that the etching rates are highly dependent on the physico-chemical properties of the samples.

## CONCLUSION

Electrospray droplet impact/secondary ion mass spectrometry (EDI/SIMS) was applied to the analysis of double-layer samples of polystyrene (PS)/Si and poly(9,9-di-*n*-octylfluonyl-2,7diyl) (PFO)/Si. For both samples, the mass spectra were independent of the time of EDI irradiation, indicating that the occurrence of non-selective etching, *i.e.*, graphitization is essentially avoided. After the depletion of synthetic polymer samples that had been deposited, ions originating from the Si substrate were clearly detected and the ion intensities were essentially the same as the organic fragment ions. These results clearly indicate that EDI/SIMS is applicable to the analysis of multilayer samples composed of organic and inorganic materials. The etching rates for PS and PFO were roughly estimated to be 0.6 nm/min and 0.15 nm/min, respectively, under the experimental conditions used in this study. The etching rates and spatial resolution would be much improved by narrowing the beam diameter. We recently succeeded in narrowing the beam diameter to less than 10 μm.^35)^ Analyses of multilayer samples by EDI/SIMS using a narrow beam diameter are currently underway in our laboratory.

## Notes

Mass Spectrom (Tokyo) 2023; 12(1): A0114
