# Supplementary material for: An Investigation of the Non-selective Etching of Synthetic Polymers by Electrospray Droplet Impact/Secondary Ion Mass Spectrometry (EDI/SIMS)
Source: Mass Spectrom (Tokyo). 2023 Jan 31;12(1):A0114. doi: 10.5702/massspectrometry.A0114 (PMC10209658; doi:10.5702/massspectrometry.A0114)
Supplement: Supplementary Data [file massspectrometry-12-1-A0114_s001.pdf]

Non-selective Etching of Synthetic Polymers Investigated by Electrospray Droplet Impact/Secondary Ion Mass Spectrometry (EDI/SIMS)

Kenzo Hiraoka,* Yuji Sakai, Hiroyuki Kubota, Satoshi Ninomiya, Stephanie Rankin-Turner

Supporting Information

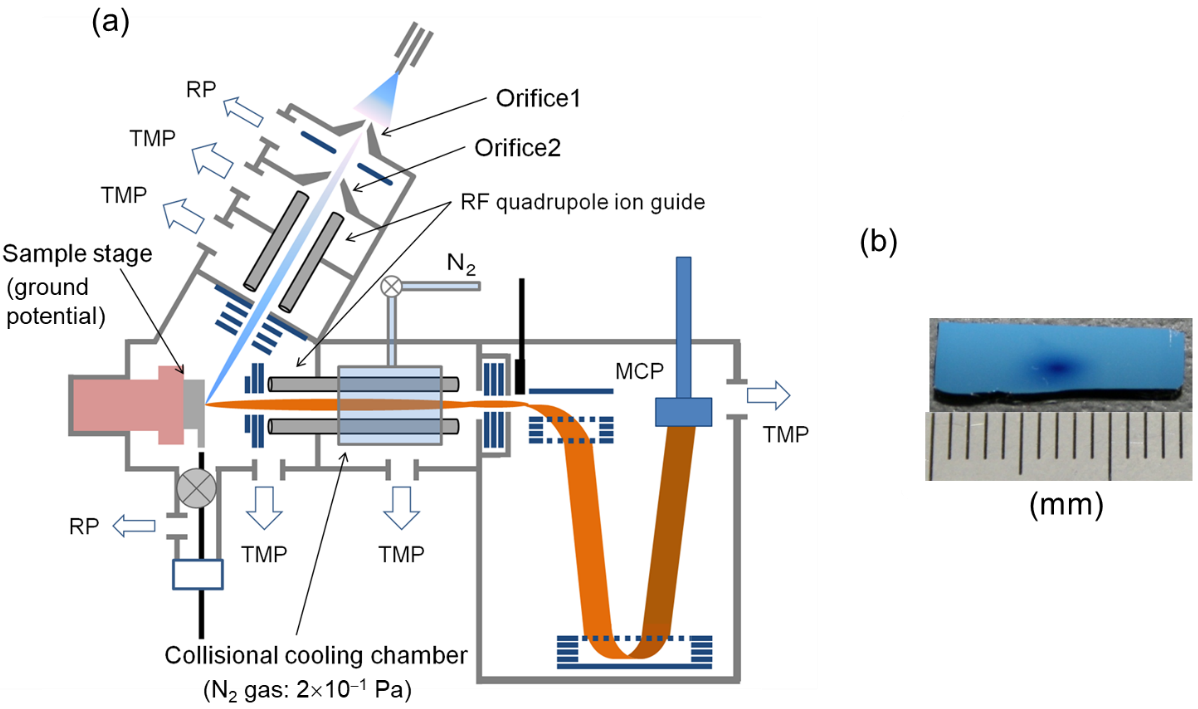

Fig. S1. (a) Schematic diagram of EDI/SIMS apparatus. (b) Silicon substrate etched by EDI for 60 min.
